# California State Veterinary Medical Association

**Published:** 1897-11

**Authors:** D. F. Fox

**Affiliations:** Secretary


### CALIFORNIA STATE VETERINARY MEDICAL ASSOCIATION.

The third quarterly meeting for the year 1897 was held in the rooms of the Baldwin Hotel, San Francisco, on September 8, 1897, commencing at 8.30 P.M. The meeting was called to order by the President, Dr. R. A. Archibald. The roll was called, and a fair attendance was present. The minutes of the previous meeting were read and approved.

Dr. Fox moved that a committee be appointed to draft resolutions of respect to the memory of our late member, Dr. P. P. Parent. The motion was carried, and Dr. Fox was appointed as said committee.

Under the head of reports of committees, Dr. Jackson, as a committee of one appointed to wait upon the San Francisco members and ask them to donate something toward defraying the expenses incurred during the last session of the Legislature, made his report. The report showed that no money had been collected since the last report; the report was accepted and the committee discharged.

Dr. H. F. Spencer, chairman of the committee appointed to draft a resolution protesting against the action of the Governor of the State of Illinois in appointing a non-graduate to the office of State Veterinarian of that State, submitted the following:

Whereas, It has come to the notice of the California State Veterinary Medical Association that Governor Tanner, Governor of the State of Illinois, has appointed a non-graduate to the office of State Veterinarian of that State; and

Whereas, It is universally admitted that Dr. Trumbower, the recent State Veterinarian of that State, was a well-qualified, painstaking, and efficient officer; and

Whereas, The action of Governor Tanner, in the appointment of a non-graduate to so important an office, establishes a pernicious example, as is evidenced by the action of the authorities of the city of Chicago in the appointment of a non-graduate to the position of veterinarian for the fire department of that city; therefore, be it

*Resolved*, That the California State Veterinary Medical Association do protest against the said appointment of a non-graduate as State Veterinarian of the State of Illinois; and be it further

*Resolved*, That the action of Dr. Joseph Hughes and Dr. J. F. Ryan, of Chicago, in resigning their positions as Assistant State Veterinarians of that State, be heartily commended by this Association; and be it further

*Resolved*, That copies of these resolutions be forwarded to Governor Tanner and to the different veterinary journals, and that they be spread upon the minutes of this Association.

Signed by H. Francis Spencer, R. A. Archibald, D. F. Fox, committee.

The resolution was unanimously adopted, and it was "so ordered."

Dr. D. F. Fox introduced the following resolution:

WHEREAS, It has pleased Almighty God, in his wise dispensation, to remove from among us our late member, Dr. P. P. Parent; be it

*Resolved*, That while we bow humbly to His will, we nevertheless feel keenly the loss we are called upon to sustain, for in our late member we had one who was devoted to his profession and to the Association, and a man who was honored and respected by the members of this Association and the entire community in which he lived; and be it further

*Resolved*, That a copy of these resolutions be spread upon the minutes of this Association and a copy be sent to the wife of the deceased. So ordered.

A communication was read from a Mr. Thomas, of San José, asking information as to the professional qualifications of Dr. F. Forrest, of San José, and whether or not he was capable of teaching his son the science of veterinary surgery intelligently. The communication was placed on file, as Mr. Thomas had already been notified that it would be wise for him to send his son to a veterinary college.

The name of Dr. P. C. Davenport was dropped from the roll of membership.

Under the head of new business, Dr. R. A. Archibald introduced the subject-matter of the appointment of a committee on diseases. He spoke at some length on the advisability of appointing such committee, the responsibility of the committee and the work it would entail upon them. Dr. Pierce also spoke on the subject; he agreed with Dr. Archibald that it would be placing a great amount of work upon a few, but at the same time he realized the benefits to be derived from such a committee. After considerable discussion by the other members present, it was decided to defer further action until the next meeting.

Nomination of officers for the ensuing year was next in order, and resulted as follows: For President, Dr. R. A. Archibald, of Oakland; for Vice-President, Dr. G. F. Faulkner, of Salinas; for Secretary, Dr. D. F. Fox, of Sacramento; for Treasurer, Dr. C. L. Megowan, of Sacramento; for Board of Examiners, no nominations. All nominations were left open until the annual meeting.

Reading of papers was postponed until the annual meeting. Several subjects were introduced for discussion, which proved to be very interesting.

Essayists for the next meeting were appointed, as follows: Dr. F. E. Pierce, of Oakland; Dr. G. F. Faulkner, of Salinas; Dr. H. A. Spencer, of San José; and Dr. C. B. Orvis, of Milton.

There being no further business before the meeting, it adjourned to meet in San Francisco, Wednesday, December 8, 1897. D. F. Fox,
Secretary.

